# Supplementary material for: Hybrid Models and Biological Model Reduction with PyDSTool
Source: PLoS Comput Biol. 2012 Aug 9;8(8):e1002628. doi: 10.1371/journal.pcbi.1002628 (PMC3415397; doi:10.1371/journal.pcbi.1002628)
Supplement: Text S4 — Complete source code for the PyDSTool package (version 0.88.120504). Includes API documentation and help files linking to web pages. This file is identical to the current public release on Sourceforge.net. (ZIP) [file pcbi.1002628.s004.zip › PyDSTool/html/PyDSTool.Toolbox.optimizers.line_search.backtracking_search.BacktrackingSearch-class.html]

xml version="1.0" encoding="ascii"?


PyDSTool.Toolbox.optimizers.line\_search.backtracking\_search.BacktrackingSearch


| Home | Trees | Indices | Help | PyDSTool |
| --- | --- | --- | --- | --- |

Package PyDSTool ::
Package Toolbox ::
Package optimizers ::
Package line\_search ::
Module backtracking\_search ::
Class BacktrackingSearch

[hide private]

[frames] | no frames]

# Class BacktrackingSearch

source code

```
object --+
         |
        BacktrackingSearch
```

---

The backtracking algorithm for enforcing Armijo rule


|  |  |
| --- | --- |
| Instance Methods | [hide private] |

|  |  |
| --- | --- |
| \_\_init\_\_(self, rho=0.1, alpha\_step=1.0, alpha\_factor=0.5, \*\*kwargs)  Can have : | source code |

|  |  |
| --- | --- |
| \_\_call\_\_(self, origin, function, state, \*\*kwargs)  Tries to find an acceptable candidate | source code |

**Inherited from `object`**:
`__delattr__`,
`__getattribute__`,
`__hash__`,
`__new__`,
`__reduce__`,
`__reduce_ex__`,
`__repr__`,
`__setattr__`,
`__str__`


|  |  |
| --- | --- |
| Properties | [hide private] |

**Inherited from `object`**:
`__class__`


|  |  |
| --- | --- |
| Method Details | [hide private] |

|  |  |
| --- | --- |
| \_\_init\_\_(self, rho=0.1, alpha\_step=1.0, alpha\_factor=0.5, \*\*kwargs)  *(Constructor)* | source code |

Can have :

- a coefficient for the Armijo rule (rho = 0.1)
- an alpha factor to modulate the step (alpha\_step = 1.)
- an alpha factor < 1 that will decrease the step size until the
  rule is valid (alpha\_factor = 0.5)

Overrides: object.\_\_init\_\_

  


| Home | Trees | Indices | Help | PyDSTool |
| --- | --- | --- | --- | --- |

|  |  |
| --- | --- |
| Generated by Epydoc 3.0.1 on Fri May 4 15:24:09 2012 | http://epydoc.sourceforge.net |
